# Implementing Mobile HRV Biofeedback as Adjunctive Therapy During Inpatient Psychiatric Rehabilitation Facilitates Recovery of Depressive Symptoms and Enhances Autonomic Functioning Short-Term: A 1-Year Pre–Post-intervention Follow-Up Pilot Study

**DOI:** 10.3389/fnins.2020.00738

**Published:** 2020-07-21

**Authors:** Josef M. Tatschl, Sigurd M. Hochfellner, Andreas R. Schwerdtfeger

**Affiliations:** ^1^Health Psychology Unit, Institute of Psychology, University of Graz, Graz, Austria; ^2^Privatklinik St. Radegund Betriebs GmbH, St. Radegund, Austria; ^3^BioTechMed-Graz, Graz, Austria

**Keywords:** biofeedback, slow-paced breathing, depression, heart rate variability, psychiatric rehabilitation, resonance frequency, vagus nerve stimulation

## Abstract

**Objective:**

New treatment options for depression are warranted, due to high recurrence rates. Recent research indicates benefits of heart rate variability biofeedback (HRVBF) on symptom recovery and autonomic functioning in depressed individuals. Slow-paced breathing-induced amplification of vagus nerve activity is the main element of HRVBF. Thus, the latter represents a safe and non-invasive complementary depression treatment. However, its efficacy in patients undergoing inpatient psychiatric rehabilitation receiving highly comprehensive treatments has not been evaluated.

**Methods:**

Ninety-two inpatients were randomly assigned to an intervention group (IG) or control group (CG). While the latter received the standard treatment only, adjunctive HRVBF was provided to the IG over 5 weeks. Depression severity and heart rate variability (HRV) were assessed before (pre) and after 5 weeks (post). Moreover, 1-year follow-up depression scores were available for 30 participants.

**Results:**

Although depression improved in both groups, the IG exhibited significantly larger improvements at post-assessment ($\eta_{\text{p}}^{2}$ = 0.065) and significant increases in resting LF-HRV (*d* = 0.45) and cardiorespiratory coherence (*d* = 0.61). No significant effects for RMSSD, SDNN, HF-HRV, or HR were found (*p*s > 0.05). Additionally, the IG showed a medium- to large-sized reduction in resting respiratory rate from 13.2 to 9.8 breaths per minute (*p* < 0.001, *d* = 0.86), with the CG exhibiting only a small decrease from 13.5 to 12.4 (*p* = 0.49; *d* = 0.35). While the IG exhibited significantly lower depression scores at post-assessment (*p* = 0.042, *d* = 0.79), this effect decreased during follow-up (*p* = 0.195, *d* = 0.48).

**Conclusion:**

HRVBF as adjuvant therapy during inpatient psychiatric rehabilitation facilitated depression recovery. Additionally, amplified LF-HRV as well as cardiorespiratory coherence at rest and a decrease in resting breathing frequency was observed in the HRVBF group. These findings emphasize HRVBF’s value as complementary therapy regardless of concurrent treatments. Moreover, these incremental benefits could serve as resource even after the actual training period. However, the additional antidepressant gains vanish during the long-term follow-up, indicating the need for more intense training or regular practice afterward, respectively. Thus, future studies are warranted to examine how the initial benefits of HRVBF during inpatient psychiatric rehabilitation can be preserved post discharge.

## Introduction

Depression has been identified as the leading cause of disability worldwide, affecting approximately 300 million people globally (World Health Organization, 2017; James et al., 2018). While antidepressants are still the standard treatment for depression, a debate regarding their efficacy has been emerging in recent years (Davidson, 2010; Ormel et al., 2020). A recent meta-analysis suggests only minor benefits compared to placebo treatments (Cipriani et al., 2018). Importantly, taking antidepressants seems to increase suicidality and all-cause mortality (Baldessarini et al., 2017; Maslej et al., 2017). Due to these obvious limitations of pharmacotherapy, alternative and safer treatment options are considered worthwhile. Importantly, the high recurrence rates among those affected by this debilitating disease indicate the need to complement conventional therapeutic approaches to improve depression prognosis (Burcusa and Iacono, 2007).

Of note, autonomic functioning is shifted toward increased sympathetic activity in depression (Koschke et al., 2009; Schumann et al., 2017). Importantly, autonomic activity can be reliably and non-invasively assessed through heart rate variability (HRV), which refers to the fluctuation of subsequent beat-to-beat intervals of the heart rate, with mathematical analysis of HRV permitting inferences onto the underlying vagal modulations (Berntson et al., 1997). HRV can be assessed in time-domain and frequency-domain measures (Shaffer and Ginsberg, 2017). A sensitive indicator of vagally mediated HRV is the respiratory sinus arrhythmia (RSA), which reflects the concomitant increase in heart rate with inspiration and decrease with expiration, with the exact phase relationship between respiration and heart rate depending on the breathing frequency (Berntson et al., 1997; Vaschillo et al., 2002). Additionally, the root mean square of successive differences between normal heartbeats (RMSSD) is an established marker of vagally mediated HRV (vmHRV; Schwerdtfeger et al., 2019). Noteworthily, a recent meta-analysis shows attenuated vagal functioning in depressed individuals, manifesting in decreased heart rate variability, including vmHRV (Koch et al., 2019).

The neurovisceral integration model (NIM), first postulated by Thayer and Lane (2000), provides a framework for a possible explanation regarding the link between depression and HRV. The NIM proposes that the regulation of affect, attention, and autonomic activity shares neural circuits, and therefore, vmHRV could index the efficacy of central–peripheral neural feedback loops (Thayer and Lane, 2009). Importantly, the prefrontal cortex, central to executive functions, is considered as a major effector regarding autonomic functioning, exhibiting top-down inhibition on sympathetic activity (Thayer and Lane, 2009). Thus, dysfunctional cognitions and emotions, respectively, could trigger the release of the prefrontal vagal brake, manifesting in decreased vmHRV (Thayer and Lane, 2009; Smith et al., 2017). Accordingly, perseverative cognition like rumination is associated with attenuated vmHRV (Gerteis and Schwerdtfeger, 2016; Ottaviani, 2018).

Importantly, enhancing HRV is hypothesized to increase cerebral oscillations, supposedly strengthening functional connectivity in brain areas relevant to emotion regulation, including prefrontal areas, which in return should improve mental well-being (Mather and Thayer, 2018). Hence, increasing HRV via heart rate variability biofeedback (HRVBF) could constitute an alternative treatment for alleviating depressive symptoms. HRVBF is based on the phenomenon of maximum RSA amplification occurring at a specific respiratory frequency, which on average is approximately 5.5 (0.09 Hz) breaths per minute (Vaschillo et al., 2002; Lehrer et al., 2003). Due to the cardiovascular resonance in response to this specific respiratory pattern, it has also been labeled resonant breathing (Vaschillo et al., 2006). HRVBF supposedly amplifies autonomic reflexes, like the baroreflex, ultimately enhancing autonomic functioning, which eventually increases HRV (Vaschillo et al., 2002; Lehrer et al., 2003; Lehrer and Gevirtz, 2014). Noteworthily, breathing at such a slow rate (i.e., 0.09 Hz) shifts the RSA from the high-frequency (HF; 0.15–0.4 Hz) to the low-frequency (LF; 0.04–0.15 Hz) domain of HRV, which seems primarily vagally mediated (Lehrer et al., 2003; Kromenacker et al., 2018).

Importantly, several studies have shown benefits of HRVBF on depression recovery and HRV in clinical depression (Karavidas et al., 2007; Siepmann et al., 2008; Hartogs et al., 2017; Caldwell and Steffen, 2018; Lin et al., 2019). Although compelling, small sample sizes and lack of control groups in previous research limit interpretation and long-term outcomes of HRVBF have not been evaluated yet. Thus, the present work aims to expand prior research by evaluating for the first time the short- and long-term efficacy of HRVBF in individuals undergoing inpatient psychiatric rehabilitation. Importantly, the main intent of this study was to assess the general feasibility of HRVBF to improve depressive symptoms in patients already receiving a highly comprehensive treatment program. Since aiming at elucidating HRVBF’s antidepressant efficacy on a more global level and in stationary psychiatric rehabilitation *per se*, patients with diagnoses other than depression were included.

Since inpatients are exposed to the same environmental factors during the 6-week in-clinic rehabilitation period, this provides an excellent context to investigate HRVBF’s efficacy, especially as HRV seems sensitive to external influences like diet, exercise, and even air quality (Levy et al., 1998; Haberfellner et al., 2008; Pieters et al., 2012; Kingsley and Figueroa, 2016; Young and Benton, 2018). Therefore, any HRV or depression differences occurring between the intervention group (IG) and the control group (CG) are likely due to HRVBF.

Based on the proposed benefits of HRVBF on depression recovery and autonomic functioning, we hypothesized that inpatient psychiatric rehabilitation supplemented by HRVBF will yield greater improvements in depressive symptoms and HRV than the standard treatment alone. Specifically, we expected that practicing HRVBF enhances vagal and baroreflex functioning, which should result in increased RMSSD, HF-HRV, and LF-HRV, respectively. Finally, the cumulative effect of increased vagal activity as well as improved baroreflex should manifest in improved overall variability and therefore increased SDNN. On an exploratory basis, we also evaluated whether HRVBF during rehabilitation affects 12-month recovery from depressive symptoms.

## Materials and Methods

### Ethics Statement

The authors assert that all procedures contributing to this work comply with the ethical standards of the relevant national and institutional committees on human experimentation and with the Helsinki Declaration of 1975, as revised in 2013. This study was approved by the institutional ethics committee (GZ. 39/43/63 ex 2017/18). From all participants, oral and written informed consent was obtained.

### Participants and Design

**FIGURE 1:**
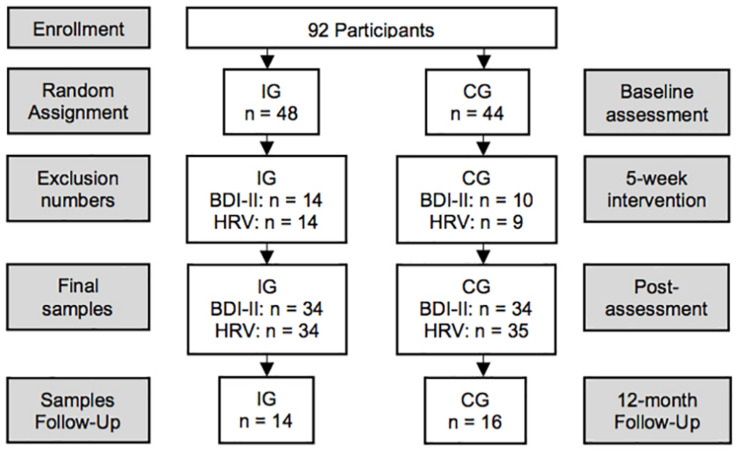
Study design. *N* = 92 in-patients enrolled in the study and were randomly assigned to the intervention (IG) or control group (CG). Due to dropouts, missing items in the BDI-II, side effects from medication, illness, artifacts in the ECG, and missing assessments, the final sample size was reduced to 68 for the depression pre–post and to 69 for the HRV pre–post analyses, respectively. Overall, 30 participants (IG = 14; CG = 16) returned the follow-up questionnaires.

Participants were recruited from a local inpatient psychiatric rehabilitation clinic, where they stay on average for 42 days. Patients taking antidepressants, anxiolytics, and other medication like anti-hypertensives or supplements, respectively, were included only if intake had been started at least 3 months prior study admission. The sampling protocol is shown in Figure 1. Patients diagnosed with a substance-use disorder were excluded. Initially, 48 participants were randomly assigned to the intervention group (IG) and 44 participants to the control group (CG). Final sample size was reduced due to dropouts (IG = 8; CG = 5), diagnosed substance-use disorder (IG = 3; CG = 2), acute illness at post-assessment (IG = 1), severe side effects due to new medication (IG = 1), missing items in the BDI-II (IG = 1; CG = 3), artifacts in the electrocardiogram (ECG; IG = 1), and missing ECG assessments (CG = 2). Thus, the depression pre–post analyses included 68 participants (IG = 34; CG = 34) aged 26–66 (*M* = 48.7; *SD* = 9.4; Table 1). Pre–post data for HRV were available from 69 participants (see Table 1). The 12-month follow-up questionnaires were returned by 30 participants (IG = 14; CG = 16).

**TABLE 1:** Demographic information for the BDI-II and HRV pre–post analyses, between groups.

	**BDI-II**			**HRV**		
	**IG**	**CG**		**IG**	**CG**	
	***n* = 34**	***n* = 34**	***p***	***n* = 34**	***n* = 35**	***p***
Age in years (*M*, *SD*)	48.5 (8.2)	48.9 (10.6)	0.858	49.03 (7.7)	49.43 (10.4)	0.857
Female sex (%)	67.6	61.8	0.612	64.7	60.0	0.687
Body mass index (*M*, *SD*)	25.6 (4.9)	27.9 (6.6)	0.105	25.7 (4.9)	27.7 (6.6)	0.165
**Primary psychiatric diagnosis ICD-10 (%)**						
Schizophrenia and schizotypal and delusional disorders F20–29 (%)	0.0	5.9	0.151	0.0	8.6	0.081
Affective disorders F30–39 (%)	76.5	73.5	0.779	76.5	71.4	0.633
Neurotic, stress-related, and somatoform disorders F40–48 (%)	23.5	20.6	0.770	23.5	20.0	0.722
Psychiatric comorbidity (%)	35.3	32.4	0.798	32.4	37.1	0.676
Burnout symptoms Z73.0 (%)	20.6	14.7	0.525	17.6	14.3	0.703
Psychotropics (%)	85.3	91.2	0.452	85.3	88.6	0.686
Psychotropics *N* (*M*, *SD*)	2.9 (1.9)	2.8 (1.5)	0.945	3.0 (2.0)	2.7 (1.5)	0.507
Antidepressants (%)	85.3	82.4	0.742	85.3	80.0	0.562
Antidepressants *N* (*M*, *SD*)	1.8 (1.1)	1.8 (1.1)	0.914	1.9 (1.2)	1.7 (1.2)	0.563
SSRI (%)	64.7	79.4	0.177	64.7	71.4	0.549
SNRI (%)	38.2	17.6	0.059	41.2	17.1	0.028
NaSSA (%)	11.8	17.6	0.493	14.7	11.4	0.686
Tricyclic (%)	5.9	0.0	0.151	5.9	0.0	0.145
Neuroleptics (%)	20.6	23.5	0.770	23.5	25.7	0.833
Atypical neuroleptics (%)	26.5	5.9	0.021	23.5	8.6	0.090
Antiepileptics (%)	5.9	26.5	0.021	5.9	28.6	0.013
Anxiolytics (%)	23.5	29.4	0.582	26.5	28.6	0.845
Anxiolytics *N* (*M*, *SD*)	0.3 (0.5)	0.3 (0.5)	0.804	0.3 (0.5)	0.3 (0.5)	0.944
Somatic comorbidity (%)	55.9	55.9	1.00	55.9	60.0	0.729
CVD (%)	17.6	17.6	1.00	17.6	20.0	0.803
Chronic pain (%)	11.8	17.6	0.493	11.8	17.1	0.526
Respiratory disease (%)	8.8	5.9	0.642	8.8	5.7	0.618
Anti-hypertensives (%)	26.5	23.5	0.779	29.4	25.7	0.731
Supplements (%)	29.4	26.5	0.787	29.4	28.6	0.939
Vitamin D^3^ (%)	11.8	20.6	0.349	11.8	20.0	0.375
Probiotics (%)	0.0	2.9	0.314	0.0	2.9	0.321
Other (%)	2.9	2.9	1.00	2.9	2.9	0.983
**Behavioral data**						
Nicotine (%)	32.4	48.5	0.178	33.3	47.1	0.252
Alcohol (%)	41.2	51.5	0.396	44.1	47.1	0.808
Aerobic training (%)	84.8	75.0	0.321	84.4	78.8	0.562
Weekly aerobic training (*M*, *SD*)	230 (192)	160 (152)	0.114	230 (195)	171 (150)	0.182
Strength (%)	6.1	6.5	0.949	6.3	12.5	0.391
Weekly strength (*M*, *SD*)	0.2 (0.7)	0.1 (0.6)	0.618	0.2 (0.7)	0.2 (0.8)	0.840
In-clinic breathing training (%)	0.0	20.6	0.005	0.0	20.0	0.006

**BDI-II, demographics between groups for depression pre–post comparisons; HRV, demographics between groups for HRV pre–post comparisons; variables tested by chi-square tests and by t-tests. CVD, cardiovascular disease. Aerobic training, total number and percentage of participants practicing aerobic exercise regularly. Weekly aerobic training, mean weekly volume of aerobic exercise in minutes. Strength, total number and percentage of participants practicing strength training regularly. Weekly strength, mean frequency of weekly strength sessions. N, total number. %, percentage. M, mean. SD, standard deviation*.*

A 2 × 2 pre–post design was applied with group (IG vs. CG) as between-subject factor, time (pre–post; post-follow-up) as within-subject factor, and depression as well as various HRV measures as dependent variables. The IG practiced HRVBF in addition to the standard treatment. The CG received standard treatment only, provided with the opportunity to receive a brief HRVBF training after the study.

### Procedure

On admission day, inpatients received an overview of the study and were assured about the confidentiality, anonymity, and possibility to withdraw from the study without negative consequences. They completed psychometric testing and two separate short-term HRV recordings, prior and after the 5-week intervention phase. After completing the baseline assessments, participants were randomly assigned to the IG or the CG, respectively (Figure 1). After post-assessment, a 12-month follow-up regarding depressive symptoms was conducted in written form. During the follow-up period, no further support was provided.

### Measures

#### Demographics and Confounders

Participants filled out questionnaires regarding demographic/control variables at study entry. At admission, diagnoses and medication including supplement intake were obtained from the patient documentations. Medications and supplements were also assessed from the demographic/control questionnaires to record potential unregistered intake. Supplements were assessed, since various substances like vitamin D^3^ or probiotics seem to have mood-altering effects (Shaffer et al., 2014; Mocking et al., 2016; Schefft et al., 2017; Nikolova et al., 2019). Additionally, the care report of every participant was reviewed to control for changes in medication, occurring illness and extraordinary incidents.

### Primary Outcomes

#### Depressive Symptoms

Depression severity was assessed with the Beck–Depression Inventory II (BDI-II) (Beck et al., 1996), which seems particularly sensitive to detect changes among psychiatric patients (Wang and Gorenstein, 2013). The BDI-II consists of 21 items and is a self-report measure, assessing cognitive, affective, and neurovegetative symptoms of depression (Beck et al., 1996; Steer and Clark, 1997). In addition to an overall depression score, BDI-II distinguishes between a cognitive and somatic–affective subscale (Huang and Chen, 2015). Cronbach’s alphas for the BDI-II overall score and the cognitive and somatic–affective subscales were 0.95, 0.91, and 0.92 at baseline; 0.95, 0.90, and 0.93 at post-assessment; and 0.95, 0.91, and 0.92 at follow-up, respectively, indicating high internal consistency (Peterson, 1994).

### Heart Rate Variability

#### HRV Data Analysis

Heart rate variability was acquired by means of the “HRV Scanner,” a one-channel ECG with a sampling rate of 500 Hz (BioSign GmbH, D-85570, Ottenhofen, Germany). The signal was obtained from two limb clamps, placed at participants’ wrists. Data analysis was performed with the HRV-Scanner Software (BioSign GmbH, D-85570, Ottenhofen, Germany). Participants completed a 3-min short-term electrocardiogram (ECG), from which time-domain and frequency-domain measures were assessed. The ECG signal was automatically controlled for artifacts by the HRV-Scanner software, and only data containing less than five percent of artifacts were included for further analyses. Additionally, the ECG was visually controlled by two experienced examiners. One participant from the IG was excluded due to excessive artifacts (baseline: 5.04%; post-assessment: 8.65%). Of note, both groups showed similar mean artifact ratios at baseline (IG: *M* = 0.16, *SD* = 0.55; CG: *M* = 0.09, *SD* = 0.28) and post-assessment (IG: *M* = 0.15, *SD* = 0.77; CG: *M* = 0.04, *SD* = 0.25).

#### HRV Measures

Heart rate variability parameters in the time domain encompass heart rate (HR), root mean square of successive differences (RMSSD), and standard deviation of RR intervals (SDNN). RMSSD is considered as a cardinal marker of parasympathetic activity and SDNN a global measure of all autonomic influences on HRV (Umetani et al., 1998; Shaffer F. et al., 2014). Frequency-domain analysis classifies HRV into low (LF; 0.04–0.15 Hz) and high frequencies (HF; 0.15–0.4 Hz), expressed as ms^2^. HF power primarily reflects parasympathetic (i.e., vagal) activity (Berntson et al., 1997). Although regarded as a marker of cardiac sympathetic control (Berntson et al., 1997), a major vagal influence on LF power is proposed (Billman, 2013; Reyes del Paso et al., 2013). Of note, during resting conditions, LF-HRV seems predominantly influenced by baroreflex and vagal activity, with only minor sympathetic contributions, compared to ambulatory settings, where sympathetic efference could be more dominant (Shaffer et al., 2014). Accordingly, Kromenacker et al. (2018) showed that increases in LF power due to slow breathing were predominantly vagally mediated.

We analyzed HR, SDNN, RMSSD, HF, and LF from the 3-min ECG recordings. Additionally, as an indicator of RSA the grade of rhythmization (GR) was calculated, which aims to quantify HRVBF success. This index integrates fluctuations of LF-HRV and HF-HRV. Specifically, changes in LF and HF are weighted against each other, with HF assigned a higher weight, thus quantifying the ratio of peak amplitude power compared to the remaining signals in the spectral analysis. This is due to the well-known phenomenon, that during states of enhanced cardiorespiratory coherence, an elevated peak and a narrower distribution of power can be observed in the spectrogram, shifting from HF to LF frequency. Therefore, GR increments correspond to an increase in the peak amplitude power, including a higher signal density centered around the peak and less power within the remaining frequencies, indicating a high RSA state. On the contrary a distribution of the power across a wider frequency range and a lower power peak, respectively, should indicate a lower GR and therefore a low RSA state. Hence, the GR aims at describing the quantity (i.e., height of amplitude) and quality of the RSA (i.e., presence of non-respiratory influences on the RSA), indicating the degree of cardiorespiratory coherence (e.g., Druschky and Druschky, 2015). It should be noted, though, that the GR is of explorative nature, since published validation studies are lacking. Frequency-domain HRV parameters were analyzed applying fast-Fourier transformation.

At the day of testing, participants were instructed to abstain from alcohol, nicotine, and exercise until HRV measurements were completed. Individuals were also instructed to fast at least 2 h prior to their appointments, as food intake potentially influences HRV (Hayano et al., 1990; Lu et al., 1999; Cornelissen et al., 2010; Romanowicz et al., 2011; Kingsley and Figueroa, 2016). Due to circadian HRV fluctuations, participants’ pre–post measurements were taken within the same 3 h of the day (Bonnemeier et al., 2003). The ECG was taken in a supine position after participants had rested for 10 min. Pre–post ECG measurements were conducted in the same climatized room.

#### Breathing Frequency

Resting breathing frequency was analyzed pre- and post-intervention from the ECG. The HRV Scanner Software analyzes respiratory rate from the ECG signal, which is highly correlated with the actual breathing rate (Schrumpf et al., 2016). Thus, ECG-derived breathing frequency has been suggested as an accurate measure of respiration (Tong et al., 2014).

### Secondary Outcomes

#### HRVBF Training Compliance

We assessed three compliance measures. First, we documented participants’ number of attended group trainings, and second, self-practice frequency was analyzed from the portable HRVBF devices. Third, an overall compliance score was calculated adding up group and self-practice frequencies.

#### HRVBF Training Performance

To measure participants’ HRVBF training performance, we assessed the relative grade of rhythmization (relGR). The relGR describes the mean achieved percentage of the set target GR (i.e., RSA amplitude required to receive a perfect feedback) during HRVBF sessions. Thus, the relGR objectifies the difficulty of the HRVBF while simultaneously measuring training success. For example, a relGR of 76 corresponds to producing on average 76% of the set target GR, while a relGR of 108 equals a mean GR, 108% of the target GR. Hence, the relGR can exceed 100% if the achieved values are higher than the set target GR, thus indicating superior performance. Additionally, respiratory rates were estimated from the biofeedback data, calculating the power peak in the frequency domains (Karlen et al., 2011).

#### Technical Details HRVBF

Heart rate variability biofeedback was delivered through a portable device named Qiu (BioSign GmbH, D-85570, Ottenhofen, Germany), which allowed participants to practice HRVBF at any time. The sphere-shaped device is battery-powered, has the size of a tennis ball, and measures heart rate by an optical sensor (i.e., photoplethysmography) at the palm, second digit, or thumb. Alternatively, an ear clip can be used to sense pulse rate. The Qiu provides the option to guide the practitioner’s BF by moving blue LED lights, which can be set individually. The Qiu records date, time, and the RR intervals of every session. Once heart rate is detected, the luminescent upper half of the Qiu visualizes the current relGR through a continuous visual feedback, which ranges from dark red (i.e., low relGR) to bright green (i.e., high relGR). Accordingly, the optical feedback displays the degree to which practitioners achieve their target GR, which can be set individually, based on the participants’ individual values. Importantly, the Qiu applies an algorithm controlling for error variance in the GR during the biofeedback, which ensures accuracy of the short feedback latency, necessary for the HRVBF.

In general, the HRVBF protocol used in this study differs from the original procedure (i.e., Lehrer et al., 2000, 2013). The original protocol assesses the precise resonance frequency with a rather time intensive procedure as a basis for the actual HRVBF (Lehrer et al., 2000, 2013). On the contrary, a 60-s deep breathing HRV test (DBT) is used to estimate the target HRV amplitude for the Qiu-HRVBF. In the DBT, participants breathe at 6 breaths per minute, which corresponds to the approximate resonance frequency, with inspiration and expiration lasting 5 s each, guided by a visual signal (Ewing and Clarke, 1982; Lehrer et al., 2003; Shields, 2009). Hence, instead of assessing the individual resonance frequency, the approximate maximum HRV amplitude is assessed from the DBT.

Precisely, the HRV Scanner software calculates the GR from the 60-s DBT, which is used by the Qiu as reference for the HRVBF. Importantly, Qiu’s target GR is set higher than the actual maximum GR amplitude achieved in the DBT. Therefore, enough margin is provided to enable practitioners to achieve their actual peak HRV during the HRVBF practice. Thus, participants have to adapt their breathing pattern in response to the visual feedback to achieve their maximum HRV (i.e., GR) amplitude. Accordingly, practitioners determine their precise resonance frequency during every training session in order to achieve a positive feedback.

### Treatments

#### Standard Treatment (ST)

The ST consisted of 240 min of daily multifaceted therapies during the week and 80 min of therapy on Saturdays. These treatments included psychotherapy, psychoeducation, music therapy, physical and exercise therapy, and relaxation methods, including progressive muscle relaxation. Importantly, inpatients have to adhere to a strict treatment curriculum, which is equal for all inpatients, with non-adherence leading to early discharge. Hence, the IG and CG were comparable regarding treatment regiments independent of the HRVBF intervention. Of note, the clinic also provided breathing training by physical therapists, as additional individual therapy. Only the CG could participate in the in-clinic breathing training to avoid any confluent effects with the HRVBF on the study outcome.

#### Details HRVBF Training Procedure

The IG received a 2-h introduction to the HRVBF, consisting of hierarchical steps: First, participants were taught nasal abdominal and pursed-lip breathing according to Lehrer et al. (2000). We emphasized nasal inspiration, as recent literature indicates improved entrainment of cerebral activity as compared to oral inspiration (Zelano et al., 2016; Herrero et al., 2018; Piarulli et al., 2018). In addition, switching from thoracic to abdominal breathing could improve vagal activation via slowly adapting stretch receptors during deep breathing (Noble and Hochman, 2019). Pursed-lip breathing is supposed to improve breathing economy through decreasing air turbulences during exhalation and mechanically dilating the airways (e.g., Lehrer et al., 2000). Also, participants were instructed to focus the mind on the Dan Tian, a supposed “energy center” in the mind–body technique of Qi Gong, allegedly located three centimeters below the navel inside the belly (Chan et al., 2008). We integrated this idea as focusing on the Dan Tian while breathing seems to facilitate slow, deep breathing, which eventually is a prerequisite to successfully modulate HRV (Lehrer et al., 2003; Chan et al., 2008). Importantly, we disentangled this concept from its dogmatic valence and instructed participants to focus on the center of their abdomen to facilitate deep breathing. Second, participants were familiarized with the Qiu. Third, they were trained to use the taught techniques to modify their breathing and to adopt the latter according to the Qiu’s visual feedback to optimize their HRV. Thus, the goal was to maximize the GR, rather than rigidly execute a specific technique. Fourth, participants received written instructions of the breathing techniques and details regarding Qiu usage, including self-practice.

The self-practice consisted of a 10-min HRVBF twice a day. Since participants had to attend the various standard treatments during the day, we recommended to do the first session in the morning and the second in the afternoon or evening, respectively. Participants were informed that they could train more HRVBF if they wanted to. Additionally, the IG was instructed to do three cycles of resonant breathing without the Qiu throughout the day, with each cycle lasting 10 breaths, trying to emulate the breathing pattern of the biofeedback-guided training. This additional practice aimed at familiarizing participants with the taught breathing techniques in order to facilitate HRVBF training. The HRVBF introduction was supplemented by one guided HRVBF session weekly (i.e., 5 sessions), consisting of approximately 35 min of HRVBF and 25 min for discussing any questions. In order to maintain training quality throughout the study period, the set target GR necessary to achieve a positive feedback was adjusted based on each individual’s progression in performance.

Because groups shared the same environment (i.e., clinic) during the study, the IG was instructed not to communicate any details about the HRVBF with the CG to avoid potential transfer effects. Importantly, participants were stressed not to share their personal HRVBF device, since all training sessions are recorded and supposed to reflect each individual’s performance and compliance, respectively.

### Statistical Analyses

Data were analyzed with SPSS 25.0 software. To compare groups regarding demographic, medical, and behavioral variables, chi-square analyses and unpaired *t*-tests were conducted. Shapiro–Wilk tests were performed to analyze distributional characteristics (Shapiro and Wilk, 1965). Accounting for skewed distributions, HRV measures were normalized using natural logarithmic transformation. Separate two-way mixed ANOVAs were performed, with group (IG, CG) as a between-subject factor and time (pre–post; post-follow-up) as a within-subject factor. Correlations were analyzed using Pearson’s product–moment correlations. As a measure of effect size, partial eta-squared ($\eta_{\text{p}}^{2}$) is reported with small, medium, and large effects represented by the values 0.01, 0.06, and 0.14, respectively (Cohen, 2013). Cohen’s *d* was reported as effect size for *t*-tests with small, medium, and large effects, represented by the values 0.2, 0.5, and 0.8, respectively (Cohen, 2013).

## Results

### Baseline Sample Characteristics

Regarding demographic and control variables, there were no significant baseline differences between the IG and CG. Both groups showed similar overall antidepressant intake and were slightly overweight with BMI values corresponding to early-stage obesity (World Health Organization, 1998; see, Table 1). However, in the IG a tendency for higher SNRI intake, significantly higher use of atypical neuroleptics, and less frequent use of antiepileptics were observed (*p*s < 0.05; Table 1).

Inpatients were diagnosed according to ICD-10 (World Health Organization, 1992). The majority was diagnosed with affective disorders (ICD-10: F30–39), followed by neurotic, stress-related, and somatoform disorders and schizophrenia and schizotypal and delusional disorders (ICD-10: F20–29), respectively. However, no information regarding the precise number of episodes in case of recurrent depression was available. Within each group, approximately one third exhibited a comorbid (two or more) disorder with about a fifth exhibiting an additional burnout (Z73.0) diagnosis (Table 1).

Importantly, for HRV pre–post analyses, demographic characteristics including both, diagnoses (including specific ICD-10 diagnoses) and control variables, were similar to the depression pre–post analyses. Only the statistical tendency for higher SNRI intake in the IG was significant (*p* < 0.05), while the less frequent use of atypical neuroleptics in the CG was non-significant (*p* > 0.05; Table 1).

Of note, groups did not differ in severity of depressive symptoms (including subscales), diagnoses, or HRV variables at baseline (*p*s > 0.05). Both groups showed moderate depression scores at baseline (Table 2).

**TABLE 2:** Paired *t*-tests by group for depression and physiological measures at baseline and post-assessment.

	**IG**				**CG**					
	***M* (*SD*)**	***M* (*SD*)**	***t***	***|d|***	***M* (*SD*)**	***M* (*SD*)**	***t***	***|d|***	***F***	**$\eta_{\text{p}}^{2}$**
	**Pre**	**Post**			**Pre**	**Post**				
BDI-II total	21.59 (12.51)	9.18 (10.13)	7.565**	1.30	19.97 (14.04)	12.5 (11.11)	4.621**	0.79	4.602*	0.065
BDI-II cog	6.77 (5.85)	2.88 (3.95)	5.468**	0.94	7.06 (6.38)	4.38 (4.56)	3.882**	0.67	1.485	0.022
BDI-II soma	13.74 (6.96)	5.82 (6.23)	7.566**	1.30	11.88 (7.78)	7.44 (6.56)	4.846**	0.83	6.230*	0.086
HR bpm	66.69 (11.11)	67.60 (11.60)	–0.593	0.10	68.50 (11.08)	69.37 (11.34)	–0.459	0.08	0.000	0.000
BF cpm	13.15 (3.63)	9.81 (2.86)	5.039**	0.86	13.52 (4.77)	12.42 (3.91)	2.039*	0.35	6.928*	0.094
lnSDNN ms	3.38 (0.45)	3.42 (0.54)	–0.432	0.07	3.42 (.44)	3.42 (.53)	–0.024	0.004	0.105	0.002
rSDNN ms	32.50 (15.18)	35.22 (21.55)			33.66 (17.06)	34.58 (18.33)				
lnRMSSD ms	3.10 (0.63)	3.00 (0.65)	1.003	0.17	2.92 (0.60)	2.93 (0.68)	–0.148	0.025	0.377	0.012
rRMSSD ms	27.02 (18.54)	24.68 (18.96)			22.52 (16.55)	24.04 (20.50)				
lnLF ms^2^	5.25 (1.19)	5.86 (1.39)	−2.636*	0.45	5.39 (1.29)	5.52 (1.40)	–0.696	0.12	2.468	0.036
rLF ms^2^	340.77 (347.43)	1051.14 (2610.37)			563.53 (1252.44)	549.93 (934.77)				
lnHF ms^2^	5.25 (1.37)	4.85 (1.37)	1.630	0.28	4.66 (1.42)	4.79 (1.56)	–0.662	0.11	2.860	0.041
rHF ms^2^	492.60 (814.66)	334.96 (587.88)			322.70 (582.84)	380.42 (696.35)				
lnGR	1.18 (0.73)	1.72 (0.96)	−3.547*	0.61	1.40 (0.91)	1.54 (0.88)	–1.291	0.22	4.736*	0.066
rGR	4.09 (2.70)	9.05 (11.30)			6.08 (6.53)	6.74 (6.74)				

Additional F-statistics for the interaction effects in the mixed ANOVAs. BDI-II total, Beck Depression Inventory II overall score; BDI-II cog, score in the cognitive subscale of the Beck Depression Inventory II; BDI-II soma, score in the somatic-affective subscale of the Beck Depression Inventory II; HR, heart rate; bpm, beats per minute; BF, breathing frequency; cpm, cycles per minute; ms, milliseconds; ms^2^, milliseconds squared; ln, natural logarithmic normalization of the data; r, raw values; lnSDNN, logarithmized standard deviation of all normal-to-normal RR intervals; lnRMSSD, logarithmized root mean square of successive differences between normal heartbeats; lnLF, logarithmized low-frequency HRV; lnHF, logarithmized high-frequency HRV; lnGR, logarithmized grade of rhythmization; *indicates p < 0.05. **indicates p < 0.001.

### The Efficacy of HRVBF on Improving Depressive Symptoms

**FIGURE 2:**
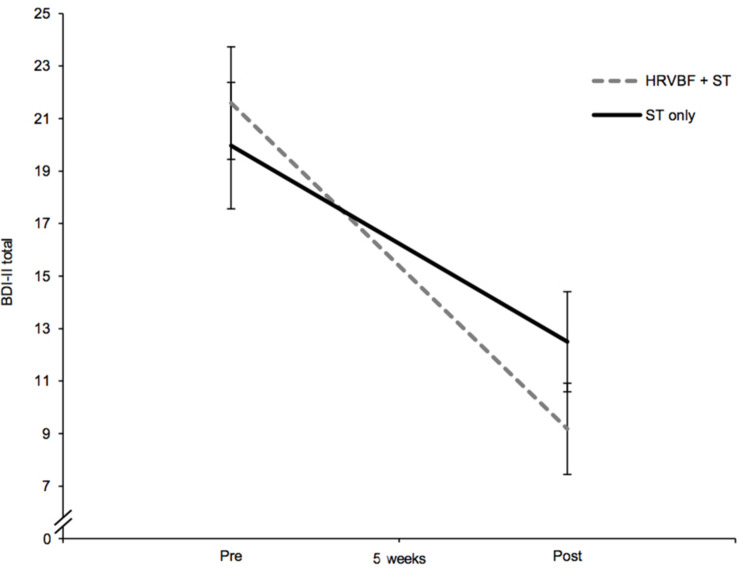
The IG, receiving the HRVBF in addition to the ST, showed a significant larger decrease in depressive symptoms over 5 weeks. BDI-II total, Beck Depression Inventory II overall score; HRVBF, heart rate variability biofeedback; ST, standard treatment. Error bars indicate ±1 SE.

Depressive symptoms decreased over the course of the 5 weeks, as evidenced by a significant main effect of time in the mixed ANOVA with a large effect size [*F*(1,66) = 74.510, *p* < 0.001, $\eta_{\text{p}}^{2}$ = 0.530]. Further, a moderating effect of group could be shown by a significant group × time interaction of medium effect size [*F*(1,66) = 4.60, *p* = 0.036, $\eta_{\text{p}}^{2}$ = 0.065; Figure 2]. Paired *t*-tests showed significant decreases in the BDI-II score of 12.4 points in the IG [*t*(33) = 7.57, *p* < 0.001, *d* = 1.30] and of 7.5 points in the CG [*t*(33) = 4.62, *p* < 0.001, *d* = 0.79; see Table 2]. Main effects for time were also found for the cognitive [*F*(1,66) = 43.92, *p* < 0.001, $\eta_{\text{p}}^{2}$ = 0.400] and somatic–affective subscales [*F*(1,66) = 78.93, *p* < 0.001, $\eta_{\text{p}}^{2}$ = 0.545] with comparably large effects. A medium-sized moderating effect of group across time could be found for somatic–affective, [*F*(1,66) = 6.23, *p* = 0.015, $\eta_{\text{p}}^{2}$ = 0.086], but not for cognitive symptoms (*p* = 0.227, $\eta_{\text{p}}^{2}$ = 0.022). Paired *t*-tests revealed a significant decrease in the somatic–affective score of 7.9 points in the IG [*t*(33) = 7.57, *p* < 0.001, *d* = 1.30] and 4.4 points in the CG [*t*(33) = 4.85, *p* < 0.001, *d* = 0.83; see Table 2], respectively. No group differences in depression (including subscales) were found at post-assessment (*p*s > 0.05).

### The Efficacy of HRVBF on Increasing Resting HRV

**FIGURE 3:**
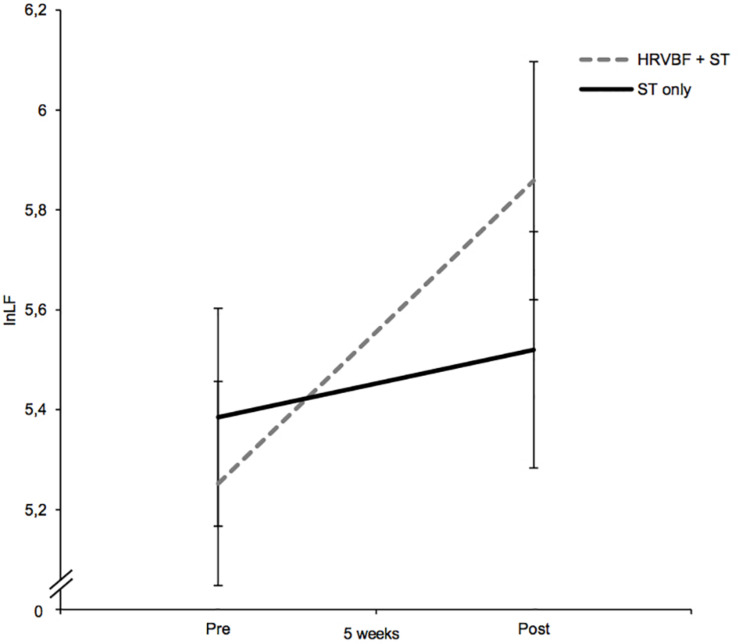
While the CL group receiving the ST only did not show any changes in lnLF, the IG (HRVBF + ST) showed an increase in lnLF after the 5-week training period. HRVBF, heart rate variability biofeedback; ST, standard treatment. Error bars indicate ±1 SE.

For lnLF, a medium-sized main effect for time could be observed [*F*(1,67) = 6.10, *p* = 0.016, $\eta_{\text{p}}^{2}$ = 0.083], suggesting increasing values from pre- to post-assessment. Albeit no significant interaction for group × time was found (*p* = 0.121, $\eta_{\text{p}}^{2}$ = 0.036), *post hoc* paired *t*-tests showed significant increases in lnLF for the IG only [*t*(33) = −2.64), *p* = 0.013, *d* = 0.45; see Figure 3] and no significant changes in the CG [*t*(34) = −0.696), *p* = 0.491, *d* = 0.12; see Table 2].

**FIGURE 4:**
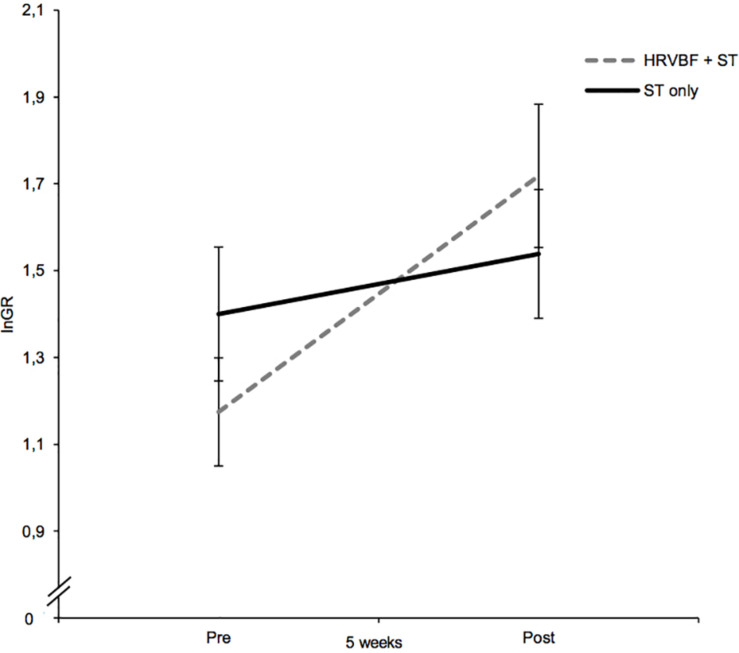
While the IG (HRVBF + ST) exhibited significant increases in lnGR, the CG (ST only) did not show any substantial changes. HRVBF, heart rate variability biofeedback; ST, standard treatment. Error bars indicate ±1 SE.

Regarding lnGR, a large-sized main effect for time [*F*(1,67) = 13.42; *p* < 0.001, $\eta_{\text{p}}^{2}$ = 0.167] and a significant interaction for group × time of medium effect size were found [*F*(1,67) = 4.74, *p* = 0.033, $\eta_{\text{p}}^{2}$ = 0.066; see Figure 4]. Paired *t*-tests evidenced a significant increase in lnGR for the IG only [*t*(33) = −3.55, *p* = 0.001, *d* = 0.61; Table 2]. No significant effects for the other HRV measures, heart rate included, were found (*p*s > 0.05).

### Effects of HRVBF on Resting Breathing Frequency

Baseline breathing rates did not differ significantly between groups (*p* = 0.717, *d* = 0.09) and were within the normal range of human respiration (IG = 13.2 vs. CG = 13.5; Yuan et al., 2013). Overall, resting breathing rate decreased, as evidenced by a large-sized main effect of time in the mixed ANOVA [*F*(1,67) = 27.16, *p* < 0.001, $\eta_{\text{p}}^{2}$ = 0.288]. A significant medium-sized interaction of time × group illustrated a moderating effect of the HRVBF on resting respiratory rate [*F*(1,67) = 6.928, *p* = 0.011, $\eta_{\text{p}}^{2}$ = 0.094]. Paired *t*-tests showed large decreases in breathing frequency for the IG, from 13.2 to 9.8 breaths per minute [*t*(33) = 5.04, *p* < 0.001, *d* = 0.86] and a small reduction from 13.5 to 12.4 for the CG [*t*(34) = 2.04, *p* = 0.049, *d* = 0.35; Table 2].

### Adherence to HRVBF Training

The mean number of attended group training sessions was 5.5 (*SD* = 0.6; range: 4–6), while the average self-practice frequency was 57 (*SD* = 26.6; range: 14–116) sessions, respectively. This corresponds to reaching a mean of 84.5% (*SD* = 34.5; range: 24.3–164.9) regarding overall target training frequency of 74 sessions (i.e., 68 self-practice sessions and 6 group trainings).

### HRVBF Training Performance

Mean relGR across sessions was 80.0%, which documents that throughout all sessions, participants achieved on average 80 percent of their individual maximum HRV peak. This indicates sufficient HRVBF training difficulty to induce potential autonomic adaptations (i.e., HRV increases). The average respiratory rate in the IG, calculated from the biofeedback data, was 5.5 (*SD* = 0.46; range: 4.7–6.8) breaths per minute, which is in line with the findings of previous research using more extensive assessment methods (Vaschillo et al., 2002; Lehrer et al., 2003). Therefore, the GR seems to provide a feasible feedback signal to foster each individual’s resonance frequency.

### Exploratory Analyses

Overall depression scores were negatively associated with lnLF (*r* = −0.346, *p* = 0.006) and lnGR (*r* = −0.319, *p* = 0.011) at pre-assessment and at post-assessment (lnLF: *r* = −0.286, *p* = 0.020; lnGR: *r* = −0.315, *p* = 0.010). None of the evaluated compliance (i.e., practice frequency) and performance measures (i.e., relGR) were associated with changes in depression or HRV, respectively (*p*s > 0.05). Also, the observed decreases in depressive symptoms including subscales were not associated with changes in any of the HRV parameters across the whole sample and within each group, respectively (*p*s > 0.05).

### 12-Month Follow-Up of Depression Recovery

From thirty participants (IG = 14; CG = 16), depression follow-up data was available. No group differences in the control variables (*p*s > 0.05) or depression severity (*p* = 0.511, *d* = 0.24) were found at baseline. A mixed ANOVA comparing depression severity between the IG and the CG from post-assessment to follow-up showed neither a significant main effect for time nor a time × group interaction (*p*s > 0.05). However, the IG exhibited significantly lower depression scores of large effect size compared to the CG (*p* = 0.042, *d* = 0.79) at post-assessment. These additional antidepressive benefits due to HRVBF decreased during the 12-month post-discharge, illustrated by slightly smaller depression differences at follow-up (*p* = 0.195, *d* = 0.48). This effect seems to originate from a visible increase in depressive symptoms within the IG during the follow-up period (*p* = 0.118, *d* = 0.48). Importantly, none of the participants from the CG completing the follow-up took part in the brief HRVBF introduction at the end of the rehabilitation.

## Discussion

This study evaluated whether HRVBF could enhance recovery of depressive symptoms and autonomic functioning in inpatients undergoing psychiatric rehabilitation. Moreover, a 12-month follow-up regarding depression trajectories was conducted, assessing the long-term sustainability of potential effects. Within 5 weeks, the IG exhibited a medium-sized, larger recovery in depressive symptoms than the CG, which appeared to be mainly driven by the comparably strong improvements in somatic–affective symptoms. However, these additional benefits gained during the treatment period vanished during the long-term follow-up. Noteworthily, toward the end of the treatment period, the IG showed medium- to large-sized amplification of LF-HRV as well as cardiorespiratory coherence (i.e., grade of rhythmization) at rest and a large reduction in resting breathing frequency, while no significant effects for RMSSD, SDNN, HF-HRV, or HR could be found. In comparison, no significant HRV changes could be observed in the CG, which, however, showed a small decrease in resting breathing rate.

Importantly, the present research complements the hitherto only randomized controlled trial by Caldwell and Steffen (2018), who showed that HRVBF facilitated depression recovery and HRV in psychotherapy patients. These effects were larger as compared to our findings, which might be attributable to differences in sample characteristics. Of note, the comparably young sample in the Caldwell and Steffen study comprised women only, who seem to respond better to depression treatment and show larger autonomic adaptions due to interventions like exercise (Genovesi et al., 2007; Donker et al., 2013). Additionally, young individuals have shown larger HRV increases in response to interventions compared to middle-aged ones (Carter et al., 2003). Noteworthily, age seems to be an important factor regarding the efficacy of HRVBF on HRV, with young samples showing more reliable increases (Lehrer et al., 2006; Alayan et al., 2019). Hence, the sample of the Caldwell and Steffen study could have been more sensitive to treatment effects regarding depression and HRV than those in the present research, who were approximately twice as old and included both sexes. It should also be mentioned that antidepressants seem to reduce HRV, with SNRIs and tricyclics having particularly unfavorable effects on vagal efferent cardiac control (Kemp et al., 2014; Alvares et al., 2016). However, SSRIs seem to attenuate vagal functioning as well, although depending on the SSRI class, with fluoxetine exhibiting the least adverse effects on HRV (Kemp et al., 2016). In this regard, it is necessary to mention that only four participants included in the HRV analyses (IG = 3; CG = 1) were taking fluoxetine. However, this was supplemented with either antipsychotics, SNRIs, additional SSRI classes, or a combination of these medications. Additionally, antipsychotics have been shown to decrease HRV as well, with atypical neuroleptics seeming especially detrimental to autonomic functioning (Agelink et al., 2001; Iwamoto et al., 2012; Linder et al., 2014). Noteworthily, the IG exhibited a high intake of SNRIs and of atypical neuroleptics, which could explain why no improvements in RMSSD, HF-HRV, or SDNN could be observed. Therefore, we suggest that the advanced age and the density of pharmacological interventions may have attenuated an increase in autonomic functioning in the IG. It is also important to note that small samples tend to exaggerate effects (Button et al., 2013). Thus, our findings may reflect HRVBF’s efficacy more accurately than the comparably smaller study of Caldwell and Steffen (2018).

Of note, this study provides first insights regarding the long-term sustainability of HRVBF-induced add-on benefits during inpatient psychiatric rehabilitation. Noteworthily, while groups showed no significant differences regarding the magnitude of depressive symptoms at baseline (*d* = 0.24), the IG compared to the CG exhibited significantly lower symptom severity of large effect size CG (*d* = 0.79) at the end of the rehabilitation period. Although these favorable antidepressive gains due to HRVBF became statistically non-significant at the 12-month follow-up assessment, these effects were still visible and of moderate size (*d* = 0.48). Seemingly, HRVBF generates unique psychophysiological benefits during the training phase, serving as additional resource even after the actual training period, which, however, appears to gradually vanish during a 12-month follow-up. Nevertheless, since we did not assess depressive symptoms and HRV at any time points between post-assessment and follow-up, no conclusions regarding psychophysiological trajectories can be drawn. Furthermore, slow-paced breathing practice during the follow-up period was not assessed, thus limiting the interpretation of the findings. However, it may be assumed that more intense training during stationary rehabilitation and/or continuing HRVBF after discharge may be necessary to maintain the initial benefits. Recently, Lin (2018) reported positive effects of a mobile-based HRVBF on autonomic balance, which could provide a useful tool to secure sustainability of the effects.

Nevertheless, the medium to large favorable effects of HRVBF shown in patients within 5 weeks of inpatient rehabilitation appear compelling and seemingly magnified the already large antidepressant effect of a well-validated, multidimensional treatment program (i.e., 25 h of weekly therapies). Moreover, the amplification of LF-HRV and GR, exclusively observed in the IG, could indicate enhanced autonomic efficacy. Importantly, under controlled resting conditions the LF-HRV seems predominantly influenced by baroreflex and vagal activity, with only minor sympathetic influences (Shaffer et al., 2014). Especially when breathing within the LF frequency range, LF-HRV reflects almost exclusively vagal efference (Kromenacker et al., 2018). Since the IG exhibited a resting breathing rate at the upper end of the LF spectrum at post-assessment, the increases in LF-HRV within the IG could be considered of vagal origin. Regarding the GR, a cautious interpretation of this measure is imperative, as validation studies are lacking. It should be noted though that participants achieved resonance breathing during the Qiu biofeedback, thus indicating the utility of the GR as a marker of cardiorespiratory coherence. Taken together, we tentatively suggest that the IG exhibited improved vagal functioning. Certainly, further studies are needed to verify of falsify this hypothesis, especially since RMSSD, a sensitive marker of vmHRV, was not affected by the resonant breathing intervention (Shaffer et al., 2014). Still, HRVBF may exhibit unique therapeutic benefits independent of concurrent treatments. Hence, these findings emphasize the distinct effect of cultivating physiological coherence through resonance breathing on human psychophysiology. Of note, a study conducted in a similar setting found no additional antidepressive benefit of a mindfulness self-compassion training (Gaiswinkler et al., 2019), despite showing antidepressant effects in a prior study (Neff and Germer, 2013). In general, breathing-based interventions seem to be of merit in improving depressive symptoms, potentially beyond conventional treatments approaches. For example, a study by Sharma et al. (2017) found that Sudarshan Kriya Yoga (SKY), a breathing-based meditation, induced large symptom improvements in depressed individuals resistant to antidepressant medication within 8 weeks. Of note, the largest reduction in depression occurred within the first 4 weeks, with small decreases during the subsequent half of the intervention period. In a further study, practicing SKY, which includes slow-paced breathing, resulted in enhanced vagal functioning in patients suffering from depression and/or anxiety, in addition to symptom reduction (Zope and Zope, 2013; Toschi-Dias et al., 2017).

Recent research indicates that the benefits of paced breathing techniques on psychological well-being could originate from breathing-induced changes in brain activation patterns. During slow-paced breathing, slowly adapting stretch receptors in the lungs are recruited and in response amplify vagal afferent input to the nucleus tractus solitarius (NTS) in the brain stem (Carr and Undem, 2003; Kubin et al., 2006). The NTS projects to cortical and subcortical areas of the brain, including prefrontal areas, the cingulate, the nucleus paraventricularis of the hypothalamus, and the amygdala, which show altered functioning in depressed individuals (Ricardo and Koh, 1978; Petrov et al., 1993; Greicius et al., 2007; Siegle et al., 2007; Bao et al., 2008; Koenigs and Grafman, 2009). Accordingly, cumulative evidence indicates that modulating respiratory patterns could entrain brain activity and, in turn, may generate a neurofunctional signature corresponding to emotional well-being (Zaccaro et al., 2018; Noble and Hochman, 2019). Hence, as hypothesized by Porges (2007), afferent vagal input, including slow-paced breathing, may aid in orchestrating emotional/psychological functioning via the cerebral susceptibility to upstream (i.e., afferent) vagal stimulation.

Indeed, these frequently suggested physiological pathways could be one origin of HRVBF’s efficacy. However, since we did not find any associations between HRV changes and improvements in depressive symptoms, including subscales, psychological mechanisms may also contribute to its potency. For example, successfully modulating Qiu’s visual feedback might foster self-efficacy, which seems decreased in depression (Bandura et al., 1999; Maeda et al., 2013). Of note, neither depression nor HRV trajectories were associated with mean relGR. Thus, the degree of participants’ exposure to negative (i.e., red), neutral (i.e., orange), or positive (i.e., green) feedback during HRVBF had no distinct effect on the main outcomes. However, the sole experience of intentionally modulating the optical feedback or feelings of relaxation independent of the actual extent may have fostered self-efficacy.

To our knowledge, this study is among the first to objectively assess whether HRVBF self-practice frequency is linked to depression or HRV changes (e.g., Karavidas et al., 2007; Siepmann et al., 2008; Caldwell and Steffen, 2018; Lin et al., 2019). Astoundingly, there were no significant associations. However, it could well be that participants generally engaged in slow-paced breathing practice independent of HRVBF, which unfortunately was not documented. Obviously, more research is necessary, targeting at the psychological and neurobiological mechanisms of the HRVBF effect. Nonetheless, considering no substantial HRV increases due to the standard treatment and the disturbed sympathetic–vagal balance in depression, our results suggest supplementing conventional therapies with HRVBF to specifically target autonomic dys/functioning (e.g., Kim et al., 2009; Koschke et al., 2009; Chien et al., 2015; Schumann et al., 2017). Moreover, the high level of practice adherence observed in the present study further supports the feasibility of HRVBF as adjunctive therapy in depressed individuals.

### Strengths and Limitations

This study has several strengths and limitations that should be mentioned. Overall, the highly standardized environment during inpatient psychiatric rehabilitation allowed us to control for various confounders, thus strengthening the validity of the findings. However, since this and prior studies were not placebo controlled, it has yet to be evaluated whether the promising antidepressive HRVBF effects are independent of a potential placebo effect. In addition, the CG did not receive a control intervention. Therefore, it could be that the additional attention due to the HRVBF group sessions may have fostered increased perceived social support and behavioral activation within the IG, thus contributing to the beneficial effects. Another positive aspect of this study is that we could objectively assess HRVBF training compliance. However, breathing practice independent of HRVBF was not documented, which may have diluted the non-findings regarding training adherence and outcome measures. Noteworthily, assessing depressive symptoms 1 year post-training constitutes a unique feature of this study as compared to previous research, thus elucidating for the first time potential long-term effects of HRVBF. On the contrary, neither HRV data at follow-up nor breathing practice during the 1-year period were obtained, thus limiting the interpretation of our results. Another factor potentially confounding the observed HRV increases is the relatively short ECG recording time of 3 min, as compared to the recommended 5 min (Berntson et al., 1997). However, cumulative research indicates the reliability of ultra-short-term HRV recordings, suggesting the validity of our findings. For example, Shaffer et al. (2016) propose that an ECG recording length of 180 s is sufficient to reliably calculate LF-HRV and HF-HRV, with 3-min recordings yielding almost identical results as 5-min measurements. Further, accurate measures of RMSSD and SDNN may be obtained from 2-min recordings (Munoz et al., 2015).

## Conclusion

The present research suggests additional benefits of HRVBF on the recovery of depressive symptoms and autonomic functioning in psychiatric rehabilitation inpatients. Thus, these findings argue for the value of HRVBF as adjunctive therapy during diverse treatment contexts, since it seemingly improves the therapeutic outcome regardless of concurrent treatment diversity and diagnosis. Importantly, the observed incremental effects could serve as resource after the training period and post-discharge, respectively. However, since the observed long-term trends did not reach significance, adequately powered follow-up studies are warranted to confirm this hypothesis and to examine ways to preserve initial therapeutic gains. Nevertheless, our findings support the implementation of HRVBF as additional intervention to foster the recovery of depressive symptoms, especially since it could provide add on-benefits, including enhanced autonomic regulation, as compared to current standard therapies. Further research is needed to examine the robustness of these findings and to control for placebo effects.

## Data Availability Statement

Due to a data policy contract with the clinic we are not allowed to provide any data to third parties. Hence, no data is available.

## Ethics Statement

The studies involving human participants were reviewed and approved by Ethics Committee University of Graz. The patients/participants provided their written informed consent to participate in this study.

## Author Contributions

JT, AS, and SH contributed to the conceptualization of the study. JT conducted the data analyses and wrote the initial draft of the manuscript. AS contributed to the manuscript revision. All authors read and approved the submitted manuscript.

## Conflict of Interest

During the conduction of the reported study, SH was employed as the medical director of the St. Radegund Betriebs GmbH. The remaining authors declare that the research was conducted in the absence of any commercial or financial relationships that could be construed as a potential conflict of interest.

## References

[B1] Agelink M. W. Majewski T. Wurthmann C. M. D. P. Lukas K. Ullrich H. Linka T. (2001). Effects of newer atypical antipsychotics on autonomic neurocardiac function: a comparison between amisulpride, olanzapine, sertindole, and clozapine. *J. Clin. Psychopharmacol.* 21 8–13. 10.1097/00004714-200102000-00003 11199953

[B2] Alayan N. Eddie D. Eller L. Bates M. E. Carmody D. P. (2019). Substance craving changes in university students receiving heart rate variability biofeedback: a longitudinal multilevel modeling approach. *Addict. Behav.* 97 35–41. 10.1016/j.addbeh.2019.05.005 31132527 PMC7454170

[B3] Alvares G. A. Quintana D. S. Hickie I. B. Guastella A. J. (2016). Autonomic nervous system dysfunction in psychiatric disorders and the impact of psychotropic medications: a systematic review and meta-analysis. *J. Psychiatry Neurosci.* 41 89–104. 10.1503/jpn.140217 26447819 PMC4764485

[B4] Baldessarini R. J. Lau W. K. Sim J. Sum M. Y. Sim K. (2017). Suicidal risks in reports of long-term controlled trials of antidepressants for major depressive disorder II. *Int. J. Neuropsychopharmacol.* 20 281–284. 10.1093/ijnp/pyw092 28003313 PMC5408981

[B5] Bandura A. Pastorelli C. Barbaranelli C. Caprara G. V. (1999). Self-efficacy pathways to childhood depression. *J. Person. Soc. Psychol.* 76 258–269. 10.1037/0022-3514.76.2.258 10074708

[B6] Bao A. M. Meynen G. Swaab D. F. (2008). The stress system in depression and neurodegeneration: focus on the human hypothalamus. *Brain Res. Rev.* 2 531–553. 10.1016/j.brainresrev.2007.04.005 17524488

[B7] Beck A. T. Steer R. A. Brown G. K. (1996). Beck depression inventory-II. *San Antonio* 78 490–498.

[B8] Berntson G. G. Bigger J. T. Eckberg D. L. Grossman P. Kaufmann P. G. Malik M. (1997). Heart rate variability: origins, methods, and interpretive caveats. *Psychophysiology* 34 623–648. 10.1111/j.1469-8986.1997.tb02140.x 9401419

[B9] Billman G. E. (2013). The LF/HF ratio does not accurately measure cardiac sympatho-vagal balance. *Front. Physiol.* 4:26. 10.3389/fphys.2013.00026 23431279 PMC3576706

[B10] Bonnemeier H. Wiegand U. K. Brandes A. Kluge N. Katus H. A. Richardt G. (2003). Circadian profile of cardiac autonomic nervous modulation in healthy subjects: differing effects of aging and gender on heart rate variability. *J. Cardiovasc. Electrophysiol.* 14 791–799. 10.1046/j.1540-8167.2003.03078.x 12890036

[B11] Burcusa S. L. Iacono W. G. (2007). Risk for recurrence in depression. *Clin. Psychol. Rev.* 27 959–985. 10.1016/j.cpr.2007.02.005 17448579 PMC2169519

[B12] Button K. S. Ioannidis J. P. Mokrysz C. Nosek B. A. Flint J. Robinson E. S. (2013). Power failure: why small sample size undermines the reliability of neuroscience. *Nat. Rev. Neurosci.* 14:365. 10.1038/nrn3475 23571845

[B13] Caldwell Y. T. Steffen P. R. (2018). Adding HRV biofeedback to psychotherapy increases heart rate variability and improves the treatment of major depressive disorder. *Int. J. Psychophysiol.* 131 96–101. 10.1016/j.ijpsycho.2018.01.001 29307738

[B14] Carr M. J. Undem B. J. (2003). Bronchopulmonary afferent nerves. *Respirology* 8 291–301. 10.1046/j.1440-1843.2003.00473.x 14528878

[B15] Carter J. B. Banister E. W. Blaber A. P. (2003). The effect of age and gender on heart rate variability after endurance training. *Med. Sci. Sports Exer.* 35 1333–1340. 10.1249/01.MSS.0000079046.01763.8F 12900687

[B16] Chan A. S. Han Y. M. Y. Cheung M.-C. (2008). Electroencephalographic (EEG) measurements of mindfulness-based triarchic body-pathway relaxation technique: a pilot study. *Appl. Psychophysiol. Biofeedb.* 33 39–47. 10.1007/s10484-008-9050-5 18214668

[B17] Chien H. C. Chung Y. C. Yeh M. L. Lee J. F. (2015). Breathing exercise combined with cognitive behavioural intervention improves sleep quality and heart rate variability in major depression. *J. Clin. Nurs.* 24 3206–3214. 10.1111/jocn.12972 26404039

[B18] Cipriani A. Furukawa T. A. Salanti G. Chaimani A. Atkinson L. Z. Ogawa Y. (2018). Comparative efficacy and acceptability of 21 antidepressant drugs for the acute treatment of adults with major depressive disorder: a systematic review and network meta-analysis. *Focus* 16, 420–429. 10.1176/appi.focus.16407 32021580 PMC6996085

[B19] Cohen J. (2013). *Statistical Power Analysis for the Behavioral Sciences.* New York: Routledge, 10.4324/9780203771587

[B20] Cornelissen V. A. Verheyden B. Aubert A. E. Fagard R. H. (2010). Effects of aerobic training intensity on resting, exercise and post-exercise blood pressure, heart rate and heart-rate variability. *J. Hum. Hyperten.* 24 175–182. 10.1038/jhh.2009.51 19554028

[B21] Davidson J. R. (2010). Major depressive disorder treatment guidelines in America and Europe. *J. Clin. Psychiatry* 71 e04–e04. 10.4088/JCP.9058se1c.04gry 20371031

[B22] Donker T. Batterham P. J. Warmerdam L. Bennett K. Bennett A. Cuijpers P. (2013). Predictors and moderators of response to internet-delivered interpersonal psychotherapy and cognitive behavior therapy for depression. *J. Affect. Disord.* 151 343–351. 10.1016/j.jad.2013.06.020 23953024

[B23] Druschky K. Druschky A. (2015). Mobile biofeedback of heart rate variability in patients with diabetic polyneuropathy: a preliminary study. *Clin. Physiol. Funct. Imaging* 35 332–333. 10.1111/cpf.12130 24438496

[B24] Ewing D. J. Clarke B. F. (1982). Diagnosis and management of diabetic autonomic neuropathy. *Br. Med. J.* 285:916. 10.1136/bmj.285.6346.916 6811067 PMC1500018

[B25] Gaiswinkler L. Kaufmann P. Pollheimer E. Ackermann A. Holasek S. Kapfhammer H. P. (2019). Mindfulness and self-compassion in clinical psychiatric rehabilitation: a clinical trial. *Mindfulness* 11 374–383. 10.1007/s12671-019-01171-1

[B26] Genovesi S. Zaccaria D. Rossi E. Valsecchi M. G. Stella A. Stramba-Badiale M. (2007). Effects of exercise training on heart rate and QT interval in healthy young individuals: are there gender differences? *Europace* 9 55–60. 10.1093/europace/eul145 17224424

[B27] Gerteis A. K. S. Schwerdtfeger A. R. (2016). When rumination counts: perceived social support and heart rate variability in daily life. *Psychophysiology* 53 1034–1043. 10.1111/psyp.12652 27137911

[B28] Greicius M. D. Flores B. H. Menon V. Glover G. H. Solvason H. B. Kenna H. (2007). Resting-state functional connectivity in major depression: abnormally increased contributions from subgenual cingulate cortex and thalamus. *Biol. Psychiatry* 62 429–437. 10.1016/j.biopsych.2006.09.020 17210143 PMC2001244

[B29] Haberfellner E. M. Jungmayr J. Grausgruber-Berner R. Grausgruber A. (2008). Medical rehabilitation of patients with mental or psychosomatic disorders in austria-findings of a catamnestic study. *Die Rehabil.* 47 164–171. 10.1055/s-2008-1076707 18553247

[B30] Hartogs B. M. Bartels-Velthuis A. A. Van der Ploeg K. Bos E. H. (2017). Heart rate variability biofeedback stress relief program for depression. *Methods Inform. Med.* 56 419–426. 10.3414/ME16-02-0033 29582913

[B31] Hayano J. Yamada M. Sakakibara Y. Fujinami T. Yokoyama K. Watanabe Y. (1990). Short-and long-term effects of cigarette smoking on heart rate variability. *Am. J. Cardiol.* 65 84–88. 10.1016/0002-9149(90)90030-5 2294686

[B32] Herrero J. L. Khuvis S. Yeagle E. Cerf M. Mehta A. D. (2018). Breathing above the brain stem: volitional control and attentional modulation in humans. *J. Neurophysiol.* 119 145–159. 10.1152/jn.00551.2017 28954895 PMC5866472

[B33] Huang C. Chen J. H. (2015). Meta-analysis of the factor structures of the beck depression inventory–II. *Assessment* 22 459–472. 10.1177/1073191114548873 25172846

[B34] Iwamoto Y. Kawanishi C. Kishida I. Furuno T. Fujibayashi M. Ishii C. (2012). Dose-dependent effect of antipsychotic drugs on autonomic nervous system activity in schizophrenia. *BMC Psychiatry* 12:199. 10.1186/1471-244X-12-199 23151241 PMC3534356

[B35] James S. L. Abate D. Abate K. H. Abay S. M. Abbafati C. Abbasi N. (2018). Global, regional, and national incidence, prevalence, and years lived with disability for 354 diseases and injuries for 195 countries and territories, 1990–2017: a systematic analysis for the global burden of disease study 2017. *Lancet* 392 1789–1858. 10.1016/S0140-6736(18)32279-7 30496104 PMC6227754

[B36] Karavidas M. K. Lehrer P. M. Vaschillo E. Vaschillo B. Marin H. Buyske S. (2007). Preliminary results of an open label study of heart rate variability biofeedback for the treatment of major depression. *Appl. Psychophysiol. Biofeedb.* 32 19–30. 10.1007/s10484-006-9029-z 17333315

[B37] Karlen W. Brouse C. J. Cooke E. Ansermino J. M. Dumont G. A. (2011). “Respiratory rate estimation using respiratory sinus arrhythmia from photoplethysmography,” in *Proceedings of the 2011 Annual International Conference of the IEEE Engineering in Medicine and Biology Society*, (Boston, MA: IEEE), 1201–1204. 10.1109/IEMBS.2011.6090282 22254531

[B38] Kemp A. H. Brunoni A. R. Santos I. S. Nunes M. A. Dantas E. M. Carvalho de Figueiredo R. (2014). Effects of depression, anxiety, comorbidity, and antidepressants on resting-state heart rate and its variability: an ELSA-Brasil cohort baseline study. *Am. J. Psychiatry* 171 1328–1334. 10.1176/appi.ajp.2014.13121605 25158141

[B39] Kemp A. H. Fráguas R. Brunoni A. R. Bittencourt M. S. Nunes M. A. Dantas E. M. (2016). Differential associations of specific selective serotonin reuptake inhibitors with resting-state heart rate and heart rate variability: implications for health and well-being. *Psychosom. Med.* 78 810–818. 10.1097/PSY.0000000000000336 27219492

[B40] Kim W. Lim S. K. Chung E. J. Woo J. M. (2009). The effect of cognitive behavior therapy-based psychotherapy applied in a forest environment on physiological changes and remission of major depressive disorder. *Psychiatry Invest.* 6 245–254. 10.4306/pi.2009.6.4.245 20140122 PMC2808793

[B41] Kingsley J. D. Figueroa A. (2016). Acute and training effects of resistance exercise on heart rate variability. *Clin. Physiol. Funct. Imaging* 36 179–187. 10.1111/cpf.12223 25524332

[B42] Koch C. Wilhelm M. Salzmann S. Rief W. Euteneuer F. (2019). A meta-analysis of heart rate variability in major depression. *Psychol. Med.* 49 1948–1957. 10.1017/S0033291719001351 31239003

[B43] Koenigs M. Grafman J. (2009). The functional neuroanatomy of depression: distinct roles for ventromedial and dorsolateral prefrontal cortex. *Behav. Brain Res.* 201 239–243. 10.1016/j.bbr.2009.03.004 19428640 PMC2680780

[B44] Koschke M. Boettger M. K. Schulz S. Berger S. Terhaar J. Voss A. (2009). Autonomy of autonomic dysfunction in major depression. *Psychosom. Med.* 71 852–860. 10.1097/PSY.0b013e3181b8bb7a 19779146

[B45] Kromenacker B. W. Sanova A. A. Marcus F. I. Allen J. J. Lane R. D. (2018). Vagal mediation of low-frequency heart rate variability during slow yogic breathing. *Psychosom. Med.* 80 581–587. 10.1097/PSY.0000000000000603 29771730

[B46] Kubin L. Alheid G. F. Zuperku E. J. McCrimmon D. R. (2006). Central pathways of pulmonary and lower airway vagal afferents. *J. Appl. Physiol.* 101 618–627. 10.1152/japplphysiol.00252.2006 16645192 PMC4503231

[B47] Lehrer P. Vaschillo E. Lu S. E. Eckberg D. Vaschillo B. Scardella A. (2006). Heart rate variability biofeedback: effects of age on heart rate variability, baroreflex gain, and asthma. *Chest* 129 278–284. 10.1378/chest.129.2.278 16478842

[B48] Lehrer P. M. Gevirtz R. (2014). Heart rate variability biofeedback: how and why does it work? *Front. Psychol.* 5:756. 10.3389/fpsyg.2014.00756 25101026 PMC4104929

[B49] Lehrer P. M. Vaschillo E. Vaschillo B. (2000). Resonant frequency biofeedback training to increase cardiac variability: rationale and manual for training. *Appl. Psychophysiol. Biofeedb.* 25 177–191. 10.1023/A:1009554825745 10999236

[B50] Lehrer P. M. Vaschillo E. Vaschillo B. Lu S. E. Eckberg D. L. Edelberg R. (2003). Heart rate variability biofeedback increases baroreflex gain and peak expiratory flow. *Psychosom. Med.* 65 796–805. 10.1097/01.PSY.0000089200.81962.19 14508023

[B51] Lehrer P. Vaschillo B. Zucker T. Graves J. Katsamanis M. Aviles M. (2013). Protocol for heart rate variability biofeedback training. *Appl. Psychophysiol. Biofeedback* 41, 98–109. 10.5298/1081-5937-41.3.08

[B52] Levy W. C. Cerqueira M. D. Harp G. D. Johannessen K. A. Abrass I. B. Schwartz R. S. (1998). Effect of endurance exercise training on heart rate variability at rest in healthy young and older men. *Am. J. Cardiol.* 82 1236–1241. 10.1016/s0002-9149(98)00611-0 9832101

[B53] Lin I. M. (2018). Effects of a cardiorespiratory synchronization training mobile application on heart rate variability and electroencephalography in healthy adults. *Int. J. Psychophysiol.* 134 168–177. 10.1016/j.ijpsycho.2018.09.005 30243751

[B54] Lin I. M. Fan S. Y. Yen C. F. Yeh Y. C. Tang T. C. Huang M. F. (2019). Heart rate variability biofeedback increased autonomic activation and improved symptoms of depression and insomnia among patients with major depression disorder. *Clin. Psychopharmacol. Neurosci.* 17:222. 10.9758/cpn.2019.17.2.222 30905122 PMC6478078

[B55] Linder J. R. Sodhi S. K. Haynes W. G. Fiedorowicz J. G. (2014). Effects of antipsychotic drugs on cardiovascular variability in participants with bipolar disorder. *Hum. Psychopharmacol.* 29, 145–151. 10.1002/hup.2380 24590543 PMC4080916

[B56] Lu C. L. Zou X. Orr W. C. Chen J. D. Z. (1999). Postprandial changes of sympathovagal balance measured by heart rate variability. *Digest. Dis. Sci.* 44 857–861. 10.1023/A:1026698800742 10219849

[B57] Maeda U. Shen B. J. Schwarz E. R. Farrell K. A. Mallon S. (2013). Self-efficacy mediates the associations of social support and depression with treatment adherence in heart failure patients. *Int. J. Behav. Med.* 20 88–96. 10.1007/s12529-011-9215-0 22212607

[B58] Maslej M. M. Bolker B. M. Russell M. J. Eaton K. Durisko Z. Hollon S. D. (2017). The mortality and myocardial effects of antidepressants are moderated by preexisting cardiovascular disease: a meta-analysis. *Psychother. Psychosom.* 86 268–282. 10.1159/000477940 28903117

[B59] Mather M. Thayer J. F. (2018). How heart rate variability affects emotion regulation brain networks. *Curr. Opin. Behav. Sci.* 19 98–104. 10.1016/j.cobeha.2017.12.017 29333483 PMC5761738

[B60] Mocking R. J. T. Harmsen I. Assies J. Koeter M. W. J. Ruhé H. Schene A. H. (2016). Meta-analysis and meta-regression of omega-3 polyunsaturated fatty acid supplementation for major depressive disorder. *Transl. Psychiatry* 6:e756. 10.1038/tp.2016.29 26978738 PMC4872453

[B61] Munoz M. L. van Roon A. Riese H. Thio C. Oostenbroek E. Westrik I. (2015). Validity of (ultra-) short recordings for heart rate variability measurements. *PLoS One* 10:e0138921. 10.1371/journal.pone.0138921 26414314 PMC4586373

[B62] Neff K. D. Germer C. K. (2013). A pilot study and randomized controlled trial of the mindful self−compassion program. *J. Clin. Psychol.* 69 28–44. 10.1002/jclp.21923 23070875

[B63] Nikolova V. Zaidi S. Y. Young A. H. Cleare A. J. Stone J. M. (2019). Gut feeling: randomized controlled trials of probiotics for the treatment of clinical depression: systematic review and meta-analysis. *Ther. Adv. Psychopharmacol.* 9:2045125319859963. 10.1177/2045125319859963 31263542 PMC6595633

[B64] Noble D. J. Hochman S. (2019). Hypothesis: pulmonary afferent activity patterns during slow, deep breathing contribute to the neural induction of physiological relaxation. *Front. Physiol.* 10:1176. 10.3389/fphys.2019.01176 31572221 PMC6753868

[B65] Ormel J. Spinhoven P. de Vries Y. A. Cramer A. O. Siegle G. J. Bockting C. L. (2020). The antidepressant standoff: why it continues and how to resolve it. *Psychol. Med.* 50 177–186. 10.1017/S0033291719003295 31779735

[B66] Ottaviani C. (2018). Brain−heart interaction in perseverative cognition. *Psychophysiology* 55:e13082. 10.1111/psyp.13082 29607505

[B67] Peterson R. A. (1994). A meta-analysis of Cronbach’s coefficient alpha. *J. Consum. Res.* 21 381–391. 10.1086/209405

[B68] Petrov T. Krukoff T. L. Jhamandas J. H. (1993). Branching projections of catecholaminergic brainstem neurons to the paraventricular hypothalamic nucleus and the central nucleus of the amygdala in the rat. *Brain Res.* 609 81–92. 10.1016/0006-8993(93)90858-K 8099526

[B69] Piarulli A. Zaccaro A. Laurino M. Menicucci D. De Vito A. Bruschini L. (2018). Ultra-slow mechanical stimulation of olfactory epithelium modulates consciousness by slowing cerebral rhythms in humans. *Sci. Rep.* 8 1–17. 10.1038/s41598-018-24924-9 29700421 PMC5919905

[B70] Pieters N. Plusquin M. Cox B. Kicinski M. Vangronsveld J. Nawrot T. S. (2012). An epidemiological appraisal of the association between heart rate variability and particulate air pollution: a meta-analysis. *Heart* 98 1127–1135. 10.1136/heartjnl-2011-301505 22628541 PMC3392690

[B71] Porges S. W. (2007). The polyvagal perspective. *Biol. Psychol.* 74 116–143. 10.1016/j.biopsycho.2006.06.009 17049418 PMC1868418

[B72] Reyes del Paso G. A. Langewitz W. Mulder L. J. Van Roon A. Duschek S. (2013). The utility of low frequency heart rate variability as an index of sympathetic cardiac tone: a review with emphasis on a reanalysis of previous studies. *Psychophysiology* 50 477–487. 10.1111/psyp.12027 23445494

[B73] Ricardo J. A. Koh E. T. (1978). Anatomical evidence of direct projections from the nucleus of the solitary tract to the hypothalamus, amygdala, and other forebrain structures in the rat. *Brain Res.* 153 1–26. 10.1016/0006-8993(78)91125-3 679038

[B74] Romanowicz M. Schmidt J. E. Bostwick J. M. Mrazek D. A. Karpyak V. M. (2011). Changes in heart rate variability associated with acute alcohol consumption: current knowledge and implications for practice and research. *Alcoholism* 35 1092–1105. 10.1111/j.1530-0277.2011.01442.x 21332532

[B75] Schefft C. Kilarski L. L. Bschor T. Koehler S. (2017). Efficacy of adding nutritional supplements in unipolar depression: a systematic review and meta-analysis. *Eur. Neuropsychopharmacol.* 27 1090–1109. 10.1016/j.euroneuro.2017.07.004 28988944

[B76] Schrumpf F. Sturm M. Bausch G. Fuchs M. (2016). Derivation of the respiratory rate from directly and indirectly measured respiratory signals using autocorrelation. *Curr. Dir. Biomed. Eng.* 2 241–245. 10.1515/cdbme-2016-0054

[B77] Schumann A. Andrack C. Baer K. J. (2017). Differences of sympathetic and parasympathetic modulation in major depression. *Prog. Neuro Psychopharmacol. Biol. Psychiatry* 79 324–331. 10.1016/j.pnpbp.2017.07.009 28710030

[B78] Schwerdtfeger A. R. Schwarz G. Pfurtscheller K. Thayer J. F. Jarczok M. N. Pfurtscheller G. (2019). Heart rate variability (HRV): from brain death to resonance breathing at 6 breaths/minute. *Clin. Neurophysiol.* 131 676–693. 10.1016/j.clinph.2019.11.013 31978852

[B79] Shaffer F. Ginsberg J. P. (2017). An overview of heart rate variability metrics and norms. *Front. Public Health* 5:258. 10.3389/fpubh.2017.00258 29034226 PMC5624990

[B80] Shaffer F. McCraty R. Zerr C. L. (2014). A healthy heart is not a metronome: an integrative review of the heart’s anatomy and heart rate variability. *Front. Psychol.* 5:1040. 10.3389/fpsyg.2014.01040 25324790 PMC4179748

[B81] Shaffer F. Shearman S. Meehan Z. M. (2016). The promise of ultra-short-term (UST) heart rate variability measurements. *Biofeedback* 44 229–233. 10.5298/1081-5937-44.3.09

[B82] Shaffer J. A. Edmondson D. Wasson L. T. Falzon L. Homma K. Ezeokoli N. (2014). Vitamin D supplementation for depressive symptoms: a systematic review and meta-analysis of randomized controlled trials. *Psychosom. Med.* 76 190–196. 10.1097/PSY.0000000000000044 24632894 PMC4008710

[B83] Shapiro S. S. Wilk M. B. (1965). An analysis of variance test for normality (Complete Samples). *Biometrika* 52 591–611. 10.2307/2333709

[B84] Sharma A. Barrett M. S. Cucchiara A. J. Gooneratne N. S. Thase M. E. (2017). A breathing-based meditation intervention for patients with major depressive disorder following inadequate response to antidepressants: a randomized pilot study. *J. Clin. Psychiatry* 78 e59–e63. 10.4088/JCP.16m10819 27898207 PMC5272872

[B85] Shields R. W. (2009). Heart rate variability with deep breathing as a clinical test of cardiovagal function. *Clevel. Clin. J. Med.* 76(Suppl 2) S37–S40. 10.3949/ccjm.76.s2.08 19376980

[B86] Siegle G. J. Thompson W. Carter C. S. Steinhauer S. R. Thase M. E. (2007). Increased amygdala and decreased dorsolateral prefrontal BOLD responses in unipolar depression: related and independent features. *Biol. Psychiatry* 61 198–209. 10.1016/j.biopsych.2006.05.048 17027931

[B87] Siepmann M. Aykac V. Unterdörfer J. Petrowski K. Mueck-Weymann M. (2008). A pilot study on the effects of heart rate variability biofeedback in patients with depression and in healthy subjects. *Appl. Psychophysiol. Biofeedb.* 33 195–201. 10.1007/s10484-008-9064-z 18807175

[B88] Smith R. Thayer J. F. Khalsa S. S. Lane R. D. (2017). The hierarchical basis of neurovisceral integration. *Neurosci. Biobehav. Rev.* 75 274–296. 10.1016/j.neubiorev.2017.02.003 28188890

[B89] Steer R. A. Clark D. A. (1997). Psychometric characteristics of the beck depression inventory-ii with college students. *Meas. Eval. Couns. Dev.* 30:128 10.1080/07481756.1997.12068933

[B90] Thayer J. F. Lane R. D. (2000). A model of neurovisceral integration in emotion regulation and dysregulation. *J. Affect. Disord.* 61 201–216. 10.1016/S0165-0327(00)00338-4 11163422

[B91] Thayer J. F. Lane R. D. (2009). Claude bernard and the heart–brain connection: further elaboration of a model of neurovisceral integration. *Neurosci. Biobehav. Rev.* 33 81–88. 10.1016/j.neubiorev.2008.08.004 18771686

[B92] Tong G. M. Zhang H. C. Guo J. H. Han F. (2014). Detection of sleep apnea-hypopnea syndrome with ECG derived respiration in Chinese population. *Int. J. Clin. Exp. Med.* 7:1269. PMC4073743 24995082

[B93] Toschi-Dias E. Tobaldini E. Solbiati M. Costantino G. Sanlorenzo R. Doria S. (2017). Sudarshan Kriya Yoga improves cardiac autonomic control in patients with anxiety-depression disorders. *J. Affect. Disord.* 214 74–80. 10.1016/j.jad.2017.03.017 28285240

[B94] Umetani K. Singer D. H. McCraty R. Atkinson M. (1998). Twenty-four hour time domain heart rate variability and heart rate: relations to age and gender over nine decades. *J. Am. College Cardiol.* 31 593–601. 10.1016/S0735-1097(97)00554-8 9502641

[B95] Vaschillo E. Lehrer P. Rishe N. Konstantinov M. (2002). Heart rate variability biofeedback as a method for assessing baroreflex function: a preliminary study of resonance in the cardiovascular system. *Appl. Psychophysiol. Biofeedb.* 27 1–27. 10.1023/A:1014587304314 12001882

[B96] Vaschillo E. G. Vaschillo B. Lehrer P. M. (2006). Characteristics of resonance in heart rate variability stimulated by biofeedback. *Appl. Psychophysiol. Biofeedb.* 31 129–142. 10.1007/s10484-006-9009-3 16838124

[B97] Wang Y. P. Gorenstein C. (2013). Psychometric properties of the beck depression inventory-II: a comprehensive review. *Braz. J. Psychiatry* 35 416–431. 10.1590/1516-4446-2012-1048 24402217

[B98] World Health Organization (1992). *The ICD-10 Classification of Mental and Behavioural Disorders: Clinical Descriptions and Diagnostic Guidelines.* Geneva: World Health Organization.

[B99] World Health Organization (2017). *Depression and Other Common Mental Disorders: Global Health Estimates.* Geneva: World Health Organization, 1–24.

[B100] World Health Organization (1998). *Obesity: Preventing and Managing the Global Epidemic: Report of a WHO Consultation on Obesity, Geneva, 3-5 June 1997 (No. WHO/NUT/NCD/98.1).* Geneva: World Health Organization. 11234459

[B101] Young H. A. Benton D. (2018). Heart-rate variability: a biomarker to study the influence of nutrition on physiological and psychological health? *Behav. Pharmacol.* 29 140–151. 10.1097/fbp.0000000000000383 29543648 PMC5882295

[B102] Yuan G. Drost N. A. McIvor R. A. (2013). Respiratory rate and breathing pattern. *McMaster Univ. Med. J.* 10 23–25.

[B103] Zaccaro A. Piarulli A. Laurino M. Garbella E. Menicucci D. Neri B. (2018). How breath-control can change your life: a systematic review on psycho-physiological correlates of slow breathing. *Front. Hum. Neurosci.* 12:353. 10.3389/fnhum.2018.00353 30245619 PMC6137615

[B104] Zelano C. Jiang H. Zhou G. Arora N. Schuele S. Rosenow J. (2016). Nasal respiration entrains human limbic oscillations and modulates cognitive function. *J. Neurosci.* 36 12448–12467. 10.1523/JNEUROSCI.2586-16 27927961 PMC5148230

[B105] Zope S. A. Zope R. A. (2013). Sudarshan kriya yoga: breathing for health. *Int. J. Yoga* 6 4–10. 10.4103/0973-6131.105935 23440614 PMC3573542

